# Genetic structure of Thai rice and rice accessions obtained from the International Rice Research Institute

**DOI:** 10.1186/1939-8433-5-19

**Published:** 2012-07-24

**Authors:** Sriprapai Chakhonkaen, Keasinee Pitnjam, Wachira Saisuk, Kittipat Ukoskit, Amorntip Muangprom

**Affiliations:** 1 Laboratory of Plant Molecular Genetics, National Center for Genetic Engineering and Biotechnology, Pathum thani, 12120 Thailand; 2 Department of Biotechnology, Faculty of Science and Technology, Thammasart University, Rangsit, Pathum thani, 12120 Thailand

**Keywords:** Genetic diversity, Germplasm evaluation, InDel marker, *Oryza sativa*, Population structure, Temperature sensitive genetic male-sterile

## Abstract

**Background:**

Although the genetic structure of rice germplasm has been characterized worldwide, few studies investigated germplasm from Thailand, the world’s largest exporter of rice. Thailand and the International Rice Research Institute (IRRI) have diverse collections of rice germplasm, which could be used to develop breeding lines with desirable traits. This study aimed to investigate the level of genetic diversity and structures of Thai and selected IRRI germplasm. Understanding the genetic structure and relationships among these germplasm will be useful for parent selection used in rice breeding programs.

**Results:**

From the 98 InDel markers tested for single copy and polymorphism, 19 markers were used to evaluate 43 Thai and 57 IRRI germplasm, including improved cultivars, breeding lines, landraces, and 5 other *Oryza* species. The Thai accessions were selected from all rice ecologies such as irrigated, deep water, upland, and rainfed lowland ecosystems. The IRRI accessions were groups of germplasm having agronomic desirable traits, including temperature-sensitive genetic male sterility (TGMS), new plant type, early flowering, and biotic and abiotic stress resistances. Most of the InDel markers were genes with diverse functions. These markers produced the total of 127 alleles for all loci, with a mean of 6.68 alleles per locus, and a mean Polymorphic Information Content (PIC) of 0.440. Genetic diversity of Thai rice were 0.3665, 0.4479 and 0.3972 for improved cultivars, breeding lines, and landraces, respectively, while genetic diversity of IRRI improved and breeding lines were 0.3272 and 0.2970, respectively. Cluster, structure, and differentiation analyses showed six distinct groups: *japonica*, TGMS, deep-water, IRRI germplasm, Thai landraces and breeding lines, and other *Oryza* species.

**Conclusions:**

Thai and IRRI germplasm were significantly different. Thus, they can be used to broaden the genetic base and trait improvements. Cluster, structure, and differentiation analyses showed concordant results having six distinct groups, in agreement with their development, and ecologies.

**Electronic supplementary material:**

The online version of this article (doi:10.1186/1939-8433-5-19) contains supplementary material, which is available to authorized users.

## Background

Genetic diversity and population structure of cultivated rice (*Oryza sativa* L.) have been studied worldwide (Garris et al. [2005]; Yu et al., [2003]; Zhao et al. [2010]; Ali et al. [2011]; Chen et al., [2011]). However, only few Thai rice germplasm has been included in these studies (Garris et al. [2005]; Yu et al., [2003]), and to our knowledge, there is no report on genetic structure of Thai commercial cultivars grown in different ecologies in the country. Thus, there is a lack of information on genetic structure of Thai rice. Thailand is the world’s largest exporter of rice, and is famous for high-quality, long-grain white rice, because the breeding of Thai rice has been focused on maintaining good grain characteristics and quality. Thailand has a large collection of diverse rice germplasm, including the famous Thai jasmine rice (Chitrakon and Somrith [2003]).

In Thailand, rice ecologies can be classified as irrigated, rainfed lowland, deep water, and upland ecosystems. Rainfed lowland is the majority of rice growing area, followed by irrigated, deepwater and upland. Different ecologies also results in different amount of rice production. The highest average rice yield was from irrigated ecosystem, followed by deep water, rainfed lowland, and upland ecosystems. The average rice yield in the wet season is constant at about 2 t/ha (http://www.fao.org/). By its shape and geography, Thailand can be divided into four regions: the mountains and forests of the North; the vast rice fields of the Central Plains; the semi-arid farm lands of the Northeast plateau; and the tropical islands and long coastline of the peninsula South. Each region has different rice-growing environments. The Northern region produces 25% of the total rice production where upland rice is grown on hilly areas, while lowland rice is grown in lower valleys and some terraced fields. The Central region produces 30% of the total rice production where rice is planted almost everywhere across the area in wet season, and in dry season rice is planted to irrigated area of about 450,000 ha. The Northeastern region produces 41% of the total rice mainly rainfed lowland rice. Only a small portion of the total rice is produced from the Southern region where rice is planted in the west and east coasts of the peninsula (http://www.irri.org).

*Oryza sativa* is composed of two major subspecies, *Indica* and *Japonica* (both tropical and temperate) and several ecotypes. Several efforts have been made to assess the genetic diversity within *Oryza sativa* at both phenotypic and molecular levels. To estimate genetic diversity among *Oryza* species, several types of molecular markers, particularly simple sequence repeats (SSR), have been used (Yu et al. [2003]; Hashimoto et al. [2004]; Garris et al. [2005]; Thomson et al. [2007]; Wen et al. [2009]; Ishii et al. [2011], Zhang et al., [2011]). Polymorphisms in the SSR region are considered the results of different replications of repeated sequences, resulting in different sizes of the PCR products. However, alleles with different sequences but having the same length may yield ambiguous results of the phylogenetic analysis. Sequencing SSR products can provide clear information on the evolutionary history of these loci (Sunnucks et al. [2000]; Provan et al. [2004]). Alternatively, single-stranded conformation polymorphism (SSCP), a simple and rapid method to determine sequence variation in a large number of samples without expensive direct sequencing, was proposed to use for genotyping and mapping genetic diversity in crop plants (Kuhn et al., [2008]). SSCP is a very sensitive technique for the detection of single point mutations between different DNA fragments (Grieu et al. [2004]; Muangprom et al. [2005]). Recently, SSCP has been used in crop studies, such as marker assisted selection (Borchert and Hohe [2009]), comparative genomics (Castelblanco and Fregene [2006]), phylogenetics (Rousseau-Gueutin et al. [2009]) and fitness effects of crop QTLs (Baack et al. [2008]). Furthermore, the recent availability of rice genome sequences provides the opportunity to select genes/sequences distributed in the genome as SSCP markers.

The International Rice Research Institute (IRRI) has large collections of characterized rice germplasm. These germplasm could be used to develop breeding lines with desirable traits, and they are available to other countries. Several of IRRI germplasm have been used for improvement of rice breeding programs in Thailand. Understanding the genetic diversity and genetic relationships among Thai and IRRI germplasm is useful for parent selection to produce hybrids or to improve rice population (Moose and Mumm [2008]). Although Thailand is famous for its rice, genetic characterizations of Thai rice at the molecular level are very limited. Therefore, the aims of this study were to evaluate the level of genetic diversity and to assess genetic relationships of Thai rice germplasm, and germplasm with desirable traits obtained from IRRI.

## Results

### InDel marker development and polymorphisms of the SSCP markers

By testing the 4 selected rice accessions with the 98 InDel markers, only markers that were presented as single copy and showed polymorphism in at least 3 out of the 4 rice accessions were selected for genetic analysis. A total of 19 InDel markers were used to evaluate genetic diversity in 101 rice accessions (Table 1). These InDel markers were chosen from 9 out of 12 rice chromosomes and most of them were genes annotated with diverse functions, as listed in Table 2.

**Table 1:** **Characteristics of rice accession used in this study including their ecologies, special traits and methods for their improvement**

No.^a^	Rice Accession	Ecologies and Special traits	Methods used for their improvement^b^
1	RD7	Irrigated, Resistance to Bacterial Blight, and Moderated resistance to Yellow Orange Leaf virus	C4-63/Gow Ruang 88//Sigadis
2	RD23	Irrigated, High yielding, Resistance to Bacterial Blight and Ragged stun	RD7/IR32//RD1
3	SPR90	Irrigated, High yielding, Resistance to Blast, Bacterial Blight, Ragged stun and Brown planthopper	RD21/IR4422-98-3-6-1//RD11/RD23
4	CNT1	Irrigated, High yielding, Resistance to Ragged stun, Brown planthopper, White-backed planthopper, and Blast	IR13146-158-1/IR15314-43-2-3-3//BKN6995-16-1-1-2
5	SPR1	Irrigated, High yielding, Resistant to Ragged stun, Brown planthopper, White-backed planthopper, Blast, Bacterial Blight, Yellow Orange Leaf virus	IR25393-57-2-3/RD23//IR27316-96-3-2-2///SPRLR77205-3-2-1-1/SPRLR79134-51-2-2
6	PTT1	Irrigated, Fragrance, High yielding, Resistance to Brown planthopper, White-backed planthopper, Blast, Bacterial Blight	BKNA6-18-3-2/PTT85061-86-3-2-1
7	PSL2	Irrigated, High yielding, Resistance to Brown planthopper and White-backed planthopper	CNTLR81122-PSL-37-2-1/SPRLR81041-194-2-1//IR56
8	SRN1	Rainfed for North-East, Resistance to Blast, Bacterial Blight, Drought and Salt	IR61078/IR46329-SRN-18-2-2-2
9	NSG19	Rainfed for North-East, Early flowering, Resistance to Brown planthopper	Breeding line
10	RD15	Rainfed for North-East, Fragrance, Good cooking quality, Moderated resistance to Drought, Early flowering, Resistance to Brown spot	Irradiated KDML105
11	KTH17	Rainfed for Central plain, Good cooking quality, Moderated resistance to Gall midge	Breeding line
12	RD27	Rainfed for Central plain, Resistance to Ragged stun, sheat blight, Blast	Khao Tah Oo/Khao Tah Haeng17
13	LPT123	Rainfed for Central plain, Resistance to acidic soil, Bacterial Blight and Ragged stun	Breeding line
14	GJ	Rainfed for South, Resistance to Ragged stun, and Narrow brown spot	Breeding line
15	LDP	Rainfed for South, Resistance to salt and acidic soil, and moderated resistant to Blast	Breeding line
16	LNP	Rainfed for South, Late flowering, Good cooking quality	Breeding line
17	CPL	Rainfed for South, Good milling quality	Breeding line
18	PG56	Deep water rice, Growing well up to 5 meters deep water, Good milling quality	Breeding line
19	LMN111	Deep water rice, Growing well up to 4 meters deep water, Resistance to drought, acidic soil, and Brown spot	Breeding line
20	HT60	Deep water rice, Growing well in Central plain area where level of water is not higher than 1 meters high, Resistance to Blast and drought	Khao Nahng Nuey11/C4-63
21	PNg1	Deep water rice, Resistance to Blast in seedling stage and stem border, and Good milling quality	Composite Crosses
22	PNg	Deep water rice, Resistance to Blast and Drought	Breeding line
23	DPY	Upland rice, South, Resistance to Blast, Brown spot, Narrow brown spot, Good cooking quality	Breeding line
24	GML	Upland rice, South, Resistance to Drought, Blast and Brown spot, Narrow brown spot	Breeding line
25	JH	Upland rice, Central and North < 1000 meters above sea level, Resistance to Blast and Gall Dwarf virus	Breeding line
26	NR	Upland rice, Growing well in 1000–1400 meters above sea level, Resistance to Blast and Cold	Breeding line
27	c20878	Upland, Primitive	Landrace
28	23006	Upland, Primitive	Landrace
29	c21486	Upland, Cold resistance	Landrace
30	23702	Upland, Cold resistance	Landrace
31	KGH	Upland, Salt resistance	Landrace
32	TM	Resistance to drought	Landrace
33	KS	Resistance to flooding	Landrace
34	KH	Resistance to Blast	Landrace
35	KK	Resistance to Brown planthopper	Landrace
36	NG	Specialty type	Landrace
37	KD	Specialty type	Landrace
38	O. *rufipogon*	*Oryza* species, Progenitor of Asian rice	
39	O.*glaberrimma*	*Oryza* species, African cultivated rice	
40	O.*brachyantha*	*Oryza* species, FF genome	
41	O. *latifolia*	*Oryza* species, CCDD genome	
42	O. *officinalis*	*Oryza* species, CC genome	
43	IR 68301-11-6-4-4-3-6-6	TGMS, tms3	Breeding line
44	IR 77271-42-5-4-36	TGMS	ID24/I 69736-175-2-2-1-1
45	IR 76753-41-6-34-13	TGMS	ID24/PSB RC 64
46	IR 76761-4-3-17-34-25	TGMS	IR 68935 S/IR32364-20-1-3-2
47	IR 73834-21-26-15-25-4	TGMS	ID24/IR58025B
48	IR 75589-31-27-8-33	TGMS	ID24/IR65469-2-3-2-3-2-2
49	IR 73827-23-26-15-7	TGMS	ID24/IR64
50	tms2 KDML105	TGMS	tms2/KDML105
51	IR 1820-52-2	Resistance to Stem borer	Breeding line
52	IR4227-28-3-2	Resistance to Stem borer, alkaline	Breeding line
53	IR 1539-823-1-4	Resistance to Brown planthopper	Breeding line
54	IR 9-60	Resistance to Brown planthopper	PETA/I-GEO-TZE
55	IR 4819-77-3-2	Resistance to Brown planthopper	Breeding line
56	IR 13146-45-2-3	Resistance to Brown planthopper	Breeding line
57	IR 13564-95-1	Resistance to Brown planthopper, Bacterial Blight	Breeding line
58	IR 2035-117-3	Resistance to white-backed planthopper, Drought	Breeding line
59	IR 36	Resistance to Gall midge, Earliness, Multiple resistance	Siam 29/Chianan 8
60	IR 14632-2-3	Resistance to Blast	Breeding line
61	IR 5533-PP854-1	Resistance to Blast	Breeding line
62	IR1905-PP11-29-4-61	Resistance to Blast	IR 8/Tetep
63	IR 54	Resistance to Bacterial blight	Tangkai rotan/IR 19
64	IR 8608-298-3-1	Resistance to Bacterial blight, Tungro virus	Breeding line
65	IR 10176-24-6-2	Earliness	Breeding line
66	IR 9202-25-1-3	Earliness	Breeding line
67	IR 50	Earliness	I-geo-tze/IR 49 a
68	IR 58	Earliness	Fb 24/IR 57 a
69	IR 72	Earliness	Taichung native 1/Chianun 242
70	IR 2153--338-3	Grain Quality	Breeding line
71	IR 12-178-2-3	Grain Quality	MONG CHIM VANG A/I-GEO-TZE
72	IR 30	Multiple resistance	Fb 24/Chianung yu 280
73	IR 32	Multiple resistance	Bpi 76/Tainan 3
74	IR 29	Multiple resistance	Fb 24/Kaohsiung 68
75	IR 28	Multiple resistance	Fb 24/Taichung 172
76	IR 26	Multiple resistance	Tangkai rotan/Kaohsiung 68
77	IR 4570-83-3-3-2 (IR 48)	Multiple resistance	Breeding line
78	IR 442-2-58	Resistance to Submergence	Breeding line
79	IR 1529-430-3 (IR 43)	Resistance to Drought	Breeding line
80	IR 5853-118-5 (IR 52)	Resistance to Drought	Breeding line
81	IR5178-1-1-4	Resistance to Drought	Breeding line
82	Azucena	Resistance to Drought	Breeding line
83	Pokkali	Resistance to Salt	Unknown derivative method
84	IR 3941-14-2-2-3	Resistance to Cold	Breeding line
85	IR 32429-122-3-1-2	Resistance to Cold	Breeding line
86	IR 42015-83-3-2-2	Resistance to Cold	Breeding line
87	IR 10206-29-2	Resistance to Salt	Breeding line
88	IR 17494-32-3-1-1-3	Resistance to Salt	Breeding line
89	IR 5657-33-2	Resistance to Salt	Breeding line
90	IR 4422-98-3-6-1	Resistance to Salt	Breeding line
91	IR 4630-22-2-17	Resistance to Salt	Breeding line
92	IR 29725-21-1-3-2	Resistance to Salt	Breeding line
93	IR 13540-56-3-2-1	Resistance to Alkaline	Breeding line
94	IR 9764-45-2-2	Resistance to Alkaline	Breeding line
95	IR 8	Plant Type	Peta/Dee-geo-woo-gen
96	IR 24	Plant Type	Chianan 8/Tangkai rotan
97	IR 5	Plant Type	Peta/Tangkai rotan
98	IR 22	Plant Type	Taichung 172/Tangkai rotan
99	IR6023-10-1-1	Plant Type	Breeding line
100	KDML105	Rainfed for North and North East, Fragrance, Good cooking quality and Resistant to Salt, Drought, Acidic soil	Breeding line
101	Nipponbare	*Japonica*	Yamabiko/Sachikaze

^a^ Number 1–26 and 100, Commercial Thai rice lines; 27–37, Landraces with special trait; 38–42,The other *Oryza* species; 43–99, Germplasm with desirable traits from IRRI.

^b^ Sources : http://iris.irri.org/; Chitrakon & Somrith [2003].

**Table 2:** **Characteristics of the InDel markers including their chromosome locations and their annotations**

Genes	Sequence	Size	Tm	Position	Annotation
				**(Chromosome; Mb)**	
1gAP006530	F: ACCCCCAGCATCTCCTCGTC	261	55	1; 15.0	intergenics
	R: ACTGGGCCAGGGCTGAGTCT				(close to OsS01g26950)
1gAP003855	F: CTTGCGCGGTCGAGTAGACG	289	55	1; 25.7	intergenics
	R: AGCGGTGTGATCCCCAAAGG				(close to Os01g45310)
Os01g48270	F: AGGCAAGCATTGGAAATAGG	210	55	1; 27.6	AAA-type ATPase family
	R: TGGTAACAATCGCACCTTGA				protein, putative, expressed
Os01g57310	F: CGATGAACTGGAACACCATGA	290	57	1; 33.1	rp1-like protein, putative, expressed
	R: GGCAACAGAGCCATACTTTGA				
Os01g72550	F: CCG AGT TCA GGC GAG TGT TC	382	55	1; 42.4	OsCML19-Calmodulin-
	R: TCA TTG TTT GGC ACT CCT CG				Related calcium sensor protein
Os02g48500	F: AGGCAATGGAGCACCAAGTT	277	57	2; 29.6	hypothetical protein
	R:TTGTACTGTTGGGGTTGGCA				
Os04g22440	F: ATCCTCGATGACACCGACCT	352	57	4; 12.6	hypothetical protein
	R: TGCCCTTGGTAACTTGCTTCT				
Os04g30430	F: GCTTCTCCTGGTTGTATGC	163	52	4: 18.0	nuclear transport factor 2,
	R:AAAATAGGGAGGCAGATAGAC				putative, expressed
4gAL606639	F: TTTTGTGAAACTTGACCCTC	112	52	4; 28.8	intergenics
	R: GCGTCCATGTCTTTATTGTG				(close to OS04g48750)
5gAC137622	F: CTCGCTGTTTACTGACTGG	155	52	5; 13.8	intergenics
	R: TTTGATGTACTGCCTGCTCT				
Os06g11600	F: TGCTGTGGGGCCTCTAATGA	211	55	6; 6.1	growth regulator related
	R: TGAGACAACACCCACCCACC				protein, putative, expressed
Os06g51110	F: GAT GGC AAA CAC CAA CAG GA	340	55	6; 30.9	cyclin, putative,
	R: GAG GGT TGG TTT GCC AGT GT				expressed
Os07g26740	F: GGGGAAGCGTCGTTATGACC	259	55	7; 15.4	60 S ribosomal protein L44,
	R: CCTTGATCGGGTGCTGAGAG				putative, expressed
Os07g027000	F: GCGCACTGTGATGCAAGATG	358	55	7; 15.6	retrotransposon protein
	R: GACCTTGTCGGGATGTGCAG				putative, unclassified
Os07g27590	F: CTGTTGAAGGGGAGGAGCGT	244	55	7;16.1	retrotransposon protein,
	R: TACGGTGCACTTCGGTCGTC				putative, unclassified
Os08g41690	F: TGCGAGGATGGAGTTCTTGA	250	55	8; 26.3	expressed protein
	R: CAATCCCTTCACCAGAAGGAC				
Os08g41950	F: GTC AGC CTG AAG TGC AGC AG	418	55	8; 26.5	OsMADS7 - MADS-box
	R: CGG CAC CAC ATA TAT GCC AC				family with MIKCc type-
					box, expressed
Os09g08960	F: GGACTGAAAACACGATCGCA	280	55	9; 4.7	retrotransposon protein,
	R: TTTTGGGGATCATCATCGACT				putative, unclassified
Os11g41390	F: AAGAAAAATATCTATTGAGGAGTG	178	52	11; 24.3	hypothetical protein
	R: GGAGGACCATAAATGACGG				

The 19 InDel markers produced the total of 127 alleles for all loci, ranging from 4 alleles (Os06g11600 and 4gAL606639) to 12 alleles (Os07g27590), with the average of 6.68 (Table 3). Genetic diversity values ranged from 0.149 (Os06g51110) to 0.768 (Os04g22440), with the average of 0.476. Polymorphism information content (PIC) values ranged from 0.145 (Os06g51110) to 0.737 (Os04g22440), with the average of 0.440.

**Table 3:** **Number of unique alleles, Nei genetic diversity, heterozygosity and PIC values at each of InDel markers testing 101 rice accessions**

Markers	No. of allele	Nei Genetic diversity	Heterozygosity	PIC
1gAP006530	6	0.629	0.598	0.573
1gAP003855	5	0.506	0.409	0.468
Os01g48270	6	0.373	0.351	0.354
Os01g57310	8	0.426	0.385	0.387
Os01g72550	6	0.512	0.338	0.470
Os02g48500	7	0.251	0.207	0.240
Os04g22440	11	0.768	0.756	0.737
Os04g30430	5	0.439	0.399	0.399
4gAL606639	4	0.583	0.531	0.533
5gAC137622	7	0.692	0.662	0.650
Os06g11600	4	0.512	0.506	0.400
Os06g51110	5	0.149	0.062	0.145
Os07g26740	7	0.267	0.126	0.256
Os07g27000	6	0.343	0.187	0.325
Os07g27590	12	0.711	0.711	0.680
Os08g41690	8	0.404	0.395	0.370
Os08g41950	8	0.619	0.599	0.554
Os09g08960	5	0.343	0.291	0.324
Os11g41390	7	0.517	0.484	0.489
**Mean**	**6.68**	**0.476**	**0.421**	**0.440**
**Total**	**127**	**-**	**-**	**-**

### Genetic diversity and genetic difference among groups of populations

Using 19 SSCP InDel markers, genetic diversity of Thai rice, IRRI germplasm, and other *Oryza* species were 0.436, 0.322, and 0.547, respectively (Table 4). To determine genetic difference among the three groups, we performed AMOVA and pairwise analyses. The AMOVA results showed that 15.06% of the variation was caused by differences among groups, while the remaining 84.94% was caused by differences within groups. The pairwise *F*_*st*_ estimates among these three groups indicated that all the three groups were significantly different from each other.

**Table 4:** **Summary of polymorphisms according to groups of populations**

Samples	Sample size	Mean no. of allele	Effective number of alleles	Mean genetic diversity	Exp. Heterozygosity	No. of polymorphic loci
All	100	5.8	1.9	0.419	0.421	19
**1. Thai rice lines**	**38**	**3.2**	**2.0**	**0.436**	**0.441**	**17**
-Thai improved cultivars	11	2.3	1.8	0.367	0.383	15
-Thai breeding lines	16	2.7	2.0	0.448	0.464	17
-Thai landraces	11	2.4	1.9	0.398	0.417	17
**2. IRRI rice lines**	**57**	**3.7**	**1.7**	**0.322**	**0.324**	**18**
-IRRI improved cultivars	24	2.6	1.7	0.328	0.334	16
- IRRI breeding lines	33	3.2	1.6	0.298	0.302	18
**3. The other** ***Oryza*** **species**	**5**	**3.1**	**2.9**	**0.547**	**0.638**	**16**

Because IRRI germplasm was used to improve rice breeding in Thailand, in this study we tested for genetic diversity and genetic difference among the groups of Thai and IRRI rice samples to determine the effects of each classification, such as improved cultivars, breeding lines, and landraces. The results showed that genetic diversity of Thai rice were 0.367, 0.448 and 0.398 for improved cultivars, breeding lines, and landraces, respectively. On the other hand, genetic diversity of IRRI improved and breeding lines were 0.327 and 0.297, respectively (Table 4). To determine genetic difference among the six groups, which are Thai improved cultivars, Thai breeding lines, Thai landraces, IRRI improved cultivars, IRRI breeding lines, and the other *Oryza* species, the AMOVA and pairwise analyses were performed. The AMOVA results showed that 13.04% of the variations were caused by differences among groups, while the remaining 86.96% were caused by differences within groups. Pairwise *F*_*st*_ estimates among groups ranged from 0.029 to 0.349. There were no significant difference among Thai improved cultivars, Thai local breeding lines and Thai landraces. Similarly, there were no significant difference between IRRI improved and IRRI breeding lines. However, all Thai groups, classified as Thai improved cultivars, Thai local breeding lines and Thai landraces were significantly different from IRRI improved and IRRI breeding lines (Table 5).

**Table 5:** **Pairwise population differentiation according to groups of populations as measured by** ***F*** **st**

Populations^a^	1	2	3	4	5	6
1	0.000					
2	0.029 ^ns^	0.000				
3	0.049 ^ns^	0.038 ^ns^	0.000			
4	0.254**	0.233*	0.258**	0.000		
5	0.089*	0.153**	0.142**	0.349**	0.000	
6	0.093*	0.143**	0.180**	0.346**	0.035 ^ns^	0.000

^a^ 1)Thai improved cultivars, 2)Thai breeding lines, 3)Thai landraces, 4) The other *Oryz* a species 5)IRRI improved cultivars, and 6) IRRI breeding lines.

** Significant at *P* < 0.001, * Significant at *P* < 0.05, ^ns^ Not Significant.

### Clustering of rice accessions using SSCP InDel markers

**Figure 1:**
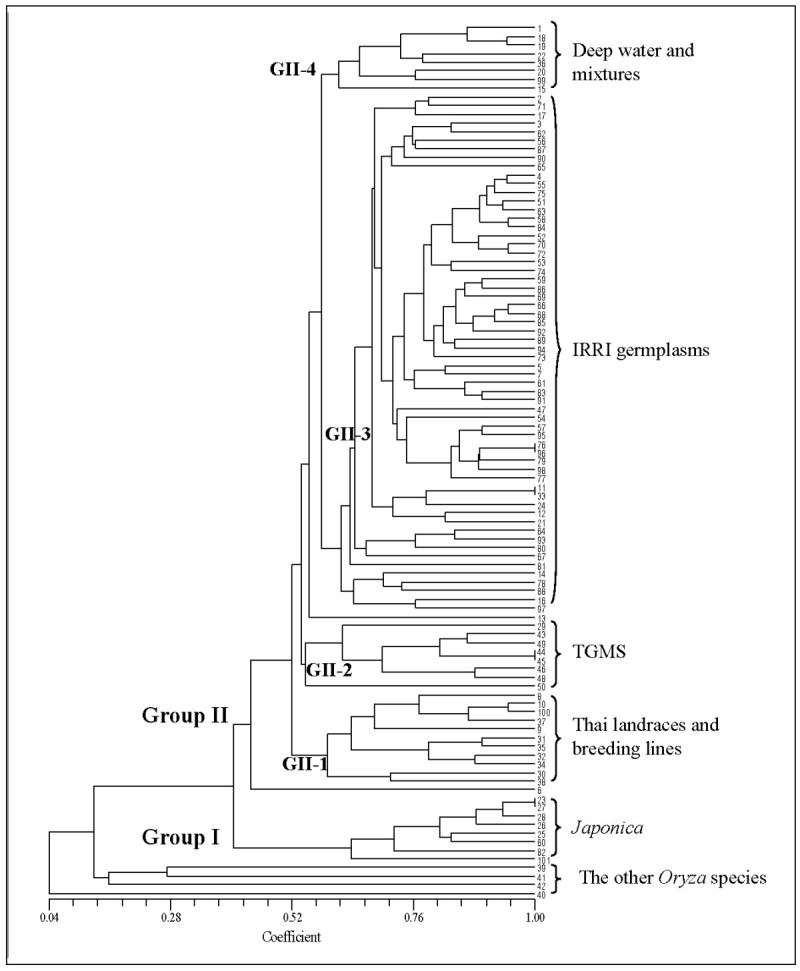
**Cluster diagram based on the Dice genetic similarity matrix by UPGMA analysis calculated from alleles of 101 rice accessions detected by 19 SSCP InDel markers; Number followed details in Table** 1 **; 1-26 and 100 are commercial Thai rice lines; 27-37 are landraces with selected traits; 38-42 are the other** ***Oryza*** **speciese; 43-99 are germplasm having desirable traits obtained from IRRI; 101 is Nipponbare.**

The UPGMA cluster diagram differentiated 4 species of other *Oryza* species and showed two major groups that correspond to the *Indica* and *Japonica* subspecies (Figure 1). Group (G) I was *Japonica* rice. Using Nipponbare as a representative for the temperate *Japonica* and Azucena (IRRI breeding line) as a representative for the tropical *Japonica*, five Thai and one IRRI accessions were grouped with *Japonica* rice lines by clustering closer to Azucena. All the Thai accessions in this group are upland rice. Group (G) II were clusters of *Indica,* which includes four sub-groups: GII-1, GII-2, GII-3, and GII-4, and additional two isolated single accessions. The GII-1 had 11 accessions which were all Thai landraces and Thai local breeding lines, except *O. rufipogon*, and one improved cultivars (No. 8, SRN1). KDML105 (No. 100), which is the famous Thai jasmine rice, was also sorted into this group by clustering with its derivative (No. 10, RD15) and a Thai landrace (No. 37, KD). The GII-2 had eight accessions that were all TGMS types, with the exception of No. 29. The GII-3 was the largest group with 60 accessions, most of which were IRRI germplasm. It should be noted that seven of the Thai improved lines and three Thai breeding lines were in this group (Figure 1). The GII-4 had eight accessions, which majority were deep water lines. The two isolated single accessions were Thai irrigated line (improved, No. 6, PTT1) and Thai rainfed central plain (breeding, No. 13, LPT123).

### Genetic structure and differentiation

**Figure 2:**
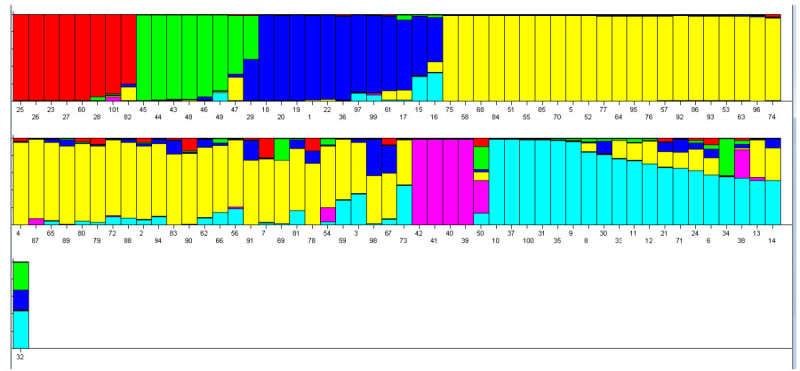
**Estimated population structure using** ***k*** **= 6; individual rice line is represented by a vertical bar broken into colored segments, with lengths in proportion to** ***Q*** **values: red,** ***Japonica*** **; green, TGMS; dark blue, Deep water rice and mixtures; yellow, IRRI germplasms; pink, the other** ***Oryza*** **species; light blue, Thai landraces and breeding lines.** The numbers marked below each line indicate the rice accession numbers as shown in details in Table 1; 1-26 and 100 are commercial Thai rice lines; 27-37 are landraces with selected traits; 38-42 are the other *Oryza* species; 43-99 are germplasm with desirable traits from IRRI; 101 is Nipponbare.

Using the data of 19 polymorphic InDel markers, the model-based method was performed to determine the genetic structure among all 101 samples. The Bayesian-based clustering method demonstrated that the highest log likelihood score was obtained when the number of populations (*k*) was equal to six. The population structure based on the *k* = 6 showed similar results to the UPGMA tree (Figure 1) by sorting rice into 6 different color-coded groups, which are *Japonica* (red), TGMS (green), deep water and mixtures (blue), IRRI germplasm (yellow), the other *Orzya* species (pink), and Thai landraces and breeding lines (light blue) (Figure 2). The first group in red, *Japonica*, had the *Q* values ranging from 0.91 to 0.989, except Azucena, No. 82 (*Q* = 0.795). All Thai rice in this group was upland rice. The second group in green contained all TGMS lines with the *Q*-values ranged from 0.87 to 0.985. The TGMS line, tms2 KDML105 (No. 50), was not in this group but it had the highest *Q* value (*Q* = 0.369) with group of the other *Oryza* species. The third group in blue had the *Q* values ranging from 0.808 to 0.985. The majority of accessions in this group were Thai deep water rice. The fourth group was the largest group (shown in yellow) with *Q* values ranging from 0.812 to 0.984, most of which were IRRI germplasm, except three Thai improved cultivars from irrigated area(No. 2, 4 and 5). The fifth group in pink contained all the other *Oryza* species with their *Q*-values higher than 0.98, except for the *O. rufipogon* (No. 38) that was grouped with Thai landraces. The last group in light blue had the *Q* values ranging from 0.811 to 0.987. Four out of the eight accessions in this group were Thai landraces (No. 30, 31, 35, 37), while the remaining accessions were all Thai commercial lines from the Northeast.

To determine genetic diversity and genetic difference in the rice samples according to population structure, we re-analyzed the data according to the population structure in Figure 2. The results showed that genetic diversity of *Japonica*, TGMS, deep water and mixtures, IRRI germplasm, the other *Oryza* species, and Thai landraces and breeding lines were 0.256, 0.239, 0.303, 0.255, 0.565, and 0.355, respectively (Table 6). The AMOVA results showed that 35.28% of the variations were caused by differences among groups, while the remaining 64.72% were caused by differences within groups. Pairwise *F*_*st*_ estimates among groups ranged from 0.204 to 0.680, and indicated that these groups were significantly different from each other.

**Table 6:** **Summary of polymorphisms for 6 groups of populations according to population structure in Figure** 2

Samples	Sample size	Mean no. of allele	Effective number of alleles	Mean genetic diversity	Exp. Heterozygosity	No. of polymorphic loci
**All**	100	5.8	1.9	0.419	0.421	19
1) *Japonica* **(** red)	8	2.1	1.5	0.256	0.273	14
2) TGMS **(** green**)**	8	1.7	1.5	0.239	0.257	11
3) Deep water **(** deep blue**)**	12	2.2	1.7	0.303	0.316	13
4) IRRI germplasms **(** Yellow)	48	2.9	1.5	0.255	0.258	15
5) The other *Oryza* species **(** Pink)	5	3.1	3.0	0.565	0.661	16
6) Thai landraces and breeding lines (blue)	19	2.6	1.8	0.355	0.365	14

## Discussion

Molecular characterization is the alternative approach to overcome several limitations of morphological characterization, which are high experimental cost, long evaluation time, and environmental effects. We reported genetic analysis in rice using groups of SSCP InDel markers, most of which were developed from putative rice genes containing short InDel. The SSCP technique can overcome the limitation of the SSR technique, which could not distinguish different DNA sequences when the DNA fragments are of the same length. DNA separation on SSCP gels is based on both size and conformation, which is determined by the DNA primary structure. Typically, single-copy amplifications showed the two-band SSCP profile, indicating a separation of sense and anti-sense strands.

Previously, SSR have been used to determine genetic variation in rice. The reported number of allele per locus, genetic diversity, and Polymorphism information content (PIC) were ranged from 4.8-14.0, 6.2-6.8 and 0.63-0.70, respectively (Ni et al. [2002]; Garris et al. [2005]; Pessoa-Filho et al. [2007]; Ram et al. [2007]). Very recenly, Ali et al. ([2011]) genotyped 409 Asian rice accessions originated from 79 countries representing all the major rice growing regions of the world using 36 SSR markers. They reported an average of 9.17 alleles per marker (range from 2 to 24), a mean genetic diversity of 0.68, and an average PIC of 0.63. In addition, Chen et al. ([2011]) studies genetic diversity of 300 rice accessions representing major geographic areas of rice growing countries in the world using 372 SNP markers. They detected 744 alleles at 372 markers, an average gene diversity of 0.358, and an average PIC of 0.285. Using 19 SSCP InDel markers to determine genetic variation in 101 rice accessions, we found that the 19 markers produced the average number of allele of 6.68, and the average PIC of 0.44. Our results on average allele per locus, genetic diversity, and PIC were higher than that reported by the study using SNP marekers (Chen et al., [2011]). Comparing to other studies using SSR, our result on the average number of allele per locus was comparable to several studies (Ni et al. [2002]; Yu et al. [2003]). However, the average PIC produced by our method was lower than that reported by the other studies using SSR, concordant with the earlier study in pearl millet, which reported the average PIC value of 0.49 by SSCP relative to the SSR value of 0.72 tested on the same genotype panel (Bertin et al. [2005]).

The subset of 19 markers selected from the total of 98 markers provided the resulting groups corresponding to the *Indica* and *Japonica* subspecies, and correlated quite well with their ecologies and their known information on their development. Previous study showed that genetic diversity was also determined in genus Orysza using 11 ISSR markers selected from 30 ISSR markers (Joshi et al. [2000]). Small numbers of markers can be used to estimate genetic diversity as shown in the earlier study when a subset of 30 markers provided the same results as using all 111 markers with the same genetic distance matrices and dendrograms (Ni et al. [2002]). Similarly, Ali et al. ([2011]) also showed that a subset of 36 SSR markers gave nearly the same results as using 169 SSR markers for population structure analysis.

Here we showed that SSCP InDel markers can be used to study plant breeding. SSCP Indel gene-based markers are very specific and can utilize their known positions in the rice genome. In addition, SNP and InDel are abundant in rice (Feltus et al. [2004]; Shen et al. [2004]; Chen et al., [2011]), which allows for the development of InDel markers even in small target areas.

Our results from the analysis of the 3 main rice groups showed that the other *Oryza* species had the highest genetic diversity, followed by Thai rice lines and IRRI germplasm. Similar to the study using Indian germplasm, the genetic diversity of the other *Oryza* species in this report is higher than that of the cultivated rice (Ram et al. [2007]). However, the genetic diversity of the other *Oryza* species used in this report (0.55) is higher than the genetic diversity of the 7 wild rice species (0.436) reported by Ram et al. ([2007]). Our results indicated that *Oryza brachyantha,* No. 40, (FF genome) is the most divergent species among the other *Oryza* species, which is also in agreement with reports from previous studies (Joshi et al. [2000], Jacquemin et al. [2009]; Lu et al. [2009]). Similarly, *Oryza officinalis* No. 42, (CC genome) and *Oryza latifolia* No. 41, (CCDD genome) are grouped together, and separated from *Oryza brachyantha*, supporting that they are in the *officinalis* complex, including diploid CC and tetraploid CCDD genomes(Joshi et al. [2000]).

The selected IRRI germplasm (including 57 rice accessions from several countries) showed lower genetic diversity than that of the Thai commercial cultivars (improved and breeding lines) and Thai landraces. The Thai breeding lines had the highest genetic diversity while the IRRI breeding lines had the lowest. Interestingly, genetic diversity of Thai landraces was lower than that of Thai breeding lines, possibly because the Thai landraces were selected only from the North and the Central parts of the country, while the breeding lines were from all over Thailand.

Our cluster analysis showed two major groups of *Oryza sativa* corresponding to the *Indica* and *Japonica* subspecies, similar to other studies (Wen et al. [2009]; Chen et al., [2011]). Both cluster and structure analyses separated a group of other *Oryza* species and showed that *O. rufipogon* (No. 38), which is considered the progenitor of *Oryza sativa*, was most related to *Oryza sativa*. Interestingly, the famous Thai jasmine rice, KDML105 (No. 100) was grouped with Thai landraces and breeding lines, and *O. rufipogon*, which indicates that the strain is native to Thailand. Our analyses support the existences of five subpopulations of *Oryza sativa*, similar to earlier studies (Garris et al. [2005]; Zhao et al. [2010], Ali et al., [2011]; Chen et al., [2011]). Several rice accessions included in our study were used in the report by Zhao et al. ([2010]), and classifications of their subpopulations were concordant. However, we did not have known samples of aus and *Group* V subpopulations (Zhao et al. [2010]) in this study, thus we can not indicate if some of our subpopulations were aus and *Group* V. Some of Thai upland rice lines and one IRRI germplasm were grouped with a well known tropical *Japonica*, Azuzena, which further suggests that these upland rice lines are also tropical *Japonica*. All irrigated rice lines, which were improved cultivars, have genetic backgrounds of IRRI germplasm supporting the results of cluster and structure analyses. The results from diversity analysis showed that the Thai germplasm were more diverse than the tested IRRI germplasm, concordant with the results from structure analysis.

Although Thailand is quite small, 513115 sq. km. (approximately the same size as France), different ecologies and geography in distinct parts of the country could have some effects on our rice diversity. The first three of the four rice ecologies: irrigated, deep water, upland, and rainfed lowland ecosystems, showed distinct groups for both cluster and structure analyses. The irrigated rice lines, planted in irrigated areas in the Central plain, were grouped together and showed similar genetic structure to IRRI germplasm. Most of the tested upland rice lines were planted in hilly areas in the North, and they were grouped with *Japonica* rice. Deep water rice lines were planted in specific areas having high levels of water in the Central plain, and they were clustered together in a distinct group. Interestingly, three out of the four rainfed lowland rice lines from the South showed genetic structure that is similar to the deep water rice. In addition, groups that were sorted by population structure also displayed significant genetic difference among them.

All tested TGMS lines controlled by 3 *tgms* genes were grouped together by cluster analysis into one sub-group of *Indica*. Results from structure analysis also supported this information, with the exception of tms2 KDML105, which showed genetic structure similar to the group of the other *Oryza* species. Results from cluster and structure analyses indicated that tms2 KDML105 (No. 50) was quite distinctive from the other TGMS lines, and it contained several different genetic fragments, including the other *Oryza* species (*Q* = 0.369), TGMS (*Q* = 0.266), Thai landraces and breeding lines (*Q* = 0.141), and IRRI germplasm (*Q* = 0.101), in agreement with its genetic background containing some part of *Japonica* genome (Pitnjam et al. [2008]), and its development (Lopez et al. [2003]). C21489 (No. 29), a cold-resistance, Thai landrace with no available information on sterility, is also clustered with the TGMS sub-group. However, the structure analysis showed that it had lower *Q* value.

## Conclusions

Our study showed the utility of SSCP InDel markers for genetic analysis of Thai and IRRI rice germplasm, as alternative to SSR markers. The resulting genetic structure and differentiation of these samples were in agreement with their ecologies and their known information on their development. The results indicated that genetic diversity of Thai commercial rice lines (improved cultivars and local breeding lines) and Thai landraces were higher than that of the tested rice germplasms obtained from IRRI. Our molecular analysis indicated that some of our cultivars were *japonica* rice, and genetic diversity is present in this set of the available germplasms. Differentiation analysis indicated that groups of IRRI germplasm were significantly different from Thai groups. Thus, these germplasm can be used to broaden the genetic base and trait improvements in rice breeding programs. Cluster and structure analyses showed concordant results having six distinct groups, and differentiation analysis supported that they were significantly different from each other. The results also indicate that TGMS lines which could be used as female parents were different from the other groups making them good candidates used to create rice hybrids having high yields. Genetic diversity and genetic relationship among these germplasms will be useful for parental line selection used in rice breeding programs and in hybrid production.

## Methods

### Plant materials

A total of 101 rice accessions, which include 43 Thai accessions, 57 germplasm obtained from the International Rice Research Institute (IRRI), and Nipponbare were used in this study (Table 1). The Thai accessions included 27 commercial cultivars, 11 landraces, and 5 other *Oryza* species. The commercial cultivars were cultivars grown in all rice ecologies through out the country such as irrigated area, rain fed low land, up land, and deep water. These commercial cultivars were 11 improved and 16 local breeding lines. The improved lines were cultivars with high yielding and/or cultivars with agronomic desirable traits. The improved cultivars were classified by their development through crossing among local cultivars and/or with other genetic sources, and their recent pedigrees were known (Table 1). The local breeding cultivars had not been bred through modern breeding procedures and their precise pedigrees were unknown. The landraces were local lines, which have not been planted for commercialization, but they have different special traits (Table 1). The other *Oryza* species included *O.glaberrimma*, African cultivated rice, and four species of wild rice: *O. rufipogon, O.branchyantha, O. latifolia, and O. officcinalis.* The rice accessions obtained from IRRI were groups of germplasm having agronomic desirable traits, including temperature-sensitive genetic male sterility (TGMS), new plant type, early flowering, and biotic and abiotic stress resistances (Table 1). Two accessions, KDML 105 (the premium jasmine rice) and Nipponbare, which are well known *Indica* and *Japonica* rice respectively, were used as controls for genetic diversity analysis and as references for control of allele sizing variation between electrophoresis runs. Nipponbare was not included in genetic diversity and pairwise analysis aimed to compare Thai and IRRI germplasm. Genetic structure of the Thai accessions is very limited, but most of them were classified by morphology as *Indica* rice. Young leaves from 3–5 mature plants of each accession grown in greenhouse were collected for DNA isolation using the CTAB method (Murray and Tompson [1980]).

### Marker development and SSCP analysis

A total of 98 InDel (Insertion-Deletion) markers were developed for the SSCP analysis, most of which were gene-based markers. Some of the 98 markers were reported by Pitnjam et al. ([2008]) and Shen et al. ([2004]). Other markers are from the 48 genes distributed over the entire rice genome, 4 genes per chromosome. According to information in the rice databases (GRAMENE http://www.gramene.org/; and http://shenghuan.shnu.edu.cn/ricemarker, last accessed on December, 2011), primers were designed using the Primer 3 program (http://biowb.sdsc.edu/CGI/BW.cgi) to amplify regions of the genes containing at least two SNPs. To increase the possibility of identifying polymorphisms between rice accessions and to facilitate polymorphism observation using SSCP, sizes of the PCR products amplified from these primers were about 200–300 bp. The markers were first used to test polymorphism among 4 rice accessions (KDML105, c20878, Nipponbare, and *O. rufipogon*) representing *indica* commercial Thai rice, landrace, *japonica* and other *Oryza* species. The markers show polymorphism in at least 3 rice accessions and shown the presence of only single copy gene were selected to test the whole set of rice samples. Details of the selected InDel markers used in this study were shown in Table 2. Amplification and polymorphism detection using SSCP were performed as previously described (Pitnjam et al. [2008]).

### Data analysis

Data were entered in the form of single-individual genotypes. Then, Program POPGENE Version 1.32 was used to calculate number of alleles per locus, average heterozygosity, and genetic diversity (Nei genetic diversity) for each maker (Yeh et al. [1999]). The informativeness of markers was assessed by calculating polymorphic information content (PIC; Botstein et al. [1980]) using PowerMarker V3.0 (Liu and Muse [2005]). The genetic diversity level within group of rice accessions was analyzed by setting up parameters as 3 {Thai rice lines, IRRI germplasm, and other *Oryza* species}, and 6 {Thai landraces, Thai improved cultivars, Thai breeding lines, IRRI improved cultivars, IRRI breeding lines, and other *Oryza* species} sources of germplasm. In addition, pairwise genetic distances between rice accessions were calculated based on the similarity coefficients following Nei and Li ([1979]). The resulting similarity matrices were used to construct dendrogram by the unweighted pair-group method with arithmetic means (UPGMA) though NTSYS-pc version 2.0 (Rohlf [1998]). Bayesian-based clustering method of analysis using the software package STRUCTURE (Prichard et al. [2000]) was performed to infer the number of populations (*k*) required for accurate data interpretation without prior information on the number of groups of accessions at which the individuals were studied. The membership probabilities (*Q*) calculated from STRUCTURE ≥ 0.80 were used to assign rice accessions to clusters. Rice accessions with membership probabilities < 0.80 for all clusters were used to detect possible genetic exchanges among groups of rice accessions. In order to describe the population genetic structure and variability among populations, the Analysis of Molecular Variance (AMOVA) was performed using the ARLEQUIN 3.11 software (Excoffier et al. [2005]). The total variance was partitioned among individuals within the same populations as well as among different populations. The permutational procedure was then used to provide significant tests for each of the hierarchical variance components based on the original distance matrices (used 1000 permutation). Wright’s inbreeding coefficient (*F*_*ST*_) values calculated by AMOVA were used as inter-population genetic distance measurements as described by Huff ([1997]).

## Authors’ original submitted files for images

Below are the links to the authors’ original submitted files for images. Authors’ original file for figure 1 Authors’ original file for figure 2
