# Supplementary figures and images for: Genetic structure of Thai rice and rice accessions obtained from the International Rice Research Institute
Source: Rice (N Y). 2012 Jul 24;5:19. doi: 10.1186/1939-8433-5-19 (PMC5520827; doi:10.1186/1939-8433-5-19)

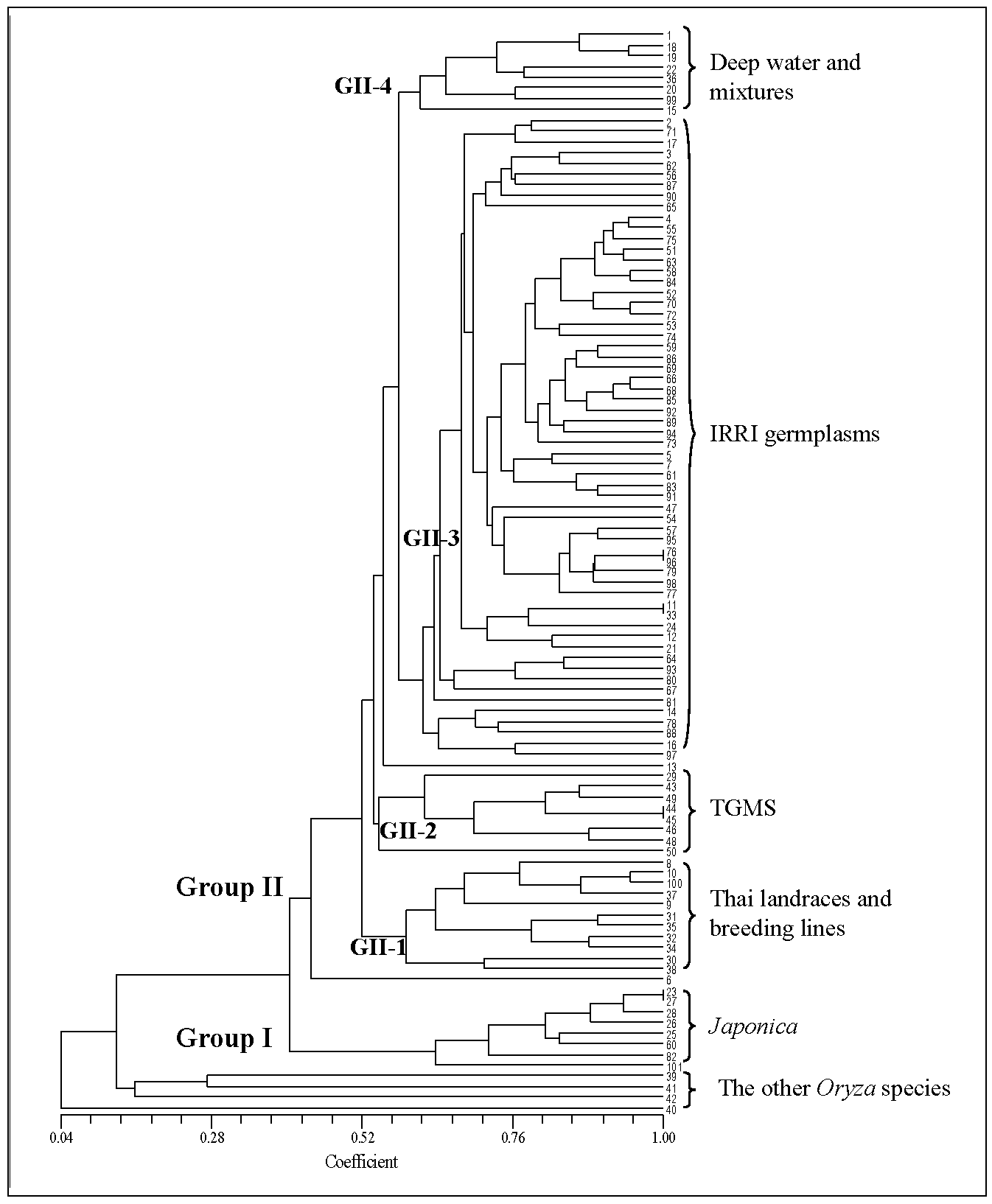

Supplement: Supplementary file 1 — Authors’ original file for figure 1 [file 12284_2012_14_MOESM1_ESM.tiff]

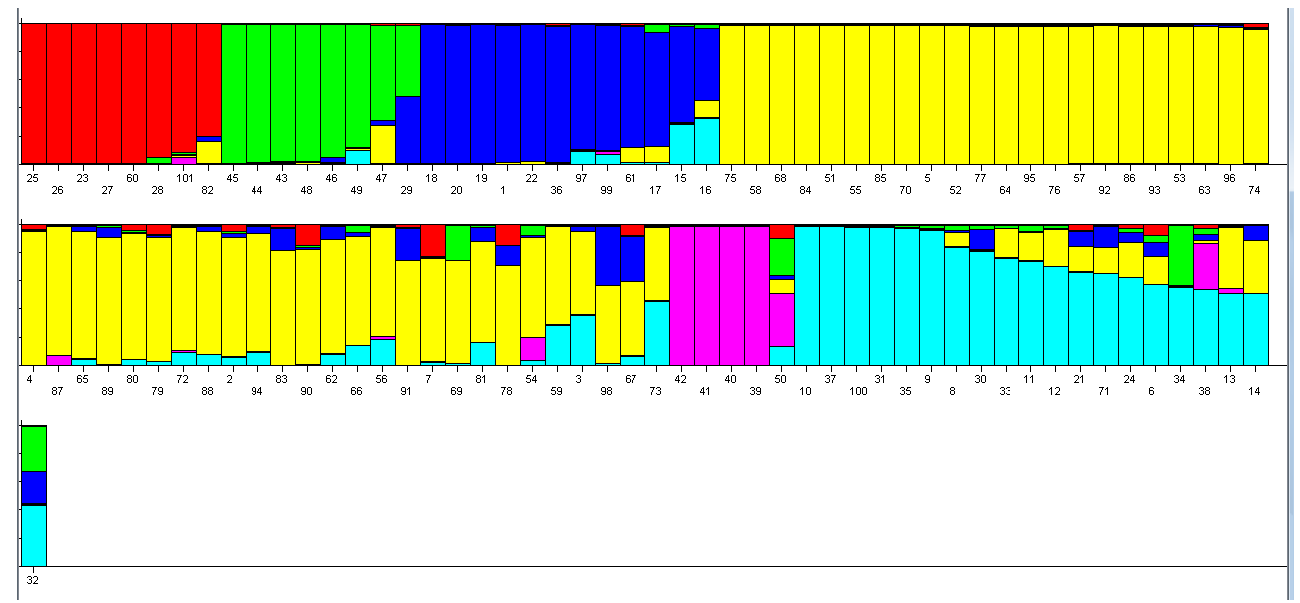

Supplement: Supplementary file 2 — Authors’ original file for figure 2 [file 12284_2012_14_MOESM2_ESM.tiff]
